# Optimization of whole slide imaging scan settings for computer vision using human lung cancer tissue

**DOI:** 10.1371/journal.pone.0309740

**Published:** 2024-09-09

**Authors:** Melvin Geubbelmans, Jari Claes, Kim Nijsten, Pascal Gervois, Simon Appeltans, Sandrina Martens, Esther Wolfs, Michiel Thomeer, Dirk Valkenborg, Christel Faes

**Affiliations:** 1 Data Science Institute, Hasselt University, Hasselt, Belgium; 2 UHasselt, Lab for Functional Imaging & Research on Stem Cells (FIERCE Lab), BIOMED, Diepenbeek, Belgium; 3 UHasselt, Limburg Clinical Research Center (LCRC), Hasselt, Belgium; 4 Department of Respiratory Medicine, Ziekenhuis Oost-Limburg, Genk, Belgium; Bayer Crop Science United States: Bayer CropScience LP, UNITED STATES OF AMERICA

## Abstract

Digital pathology has become increasingly popular for research and clinical applications. Using high-quality microscopes to produce Whole Slide Images of tumor tissue enables the discovery of insights into biological aspects invisible to the human eye. These are acquired through downstream analyses using spatial statistics and artificial intelligence. Determination of the quality and consistency of these images is needed to ensure accurate outcomes when identifying clinical and subclinical image features. Additionally, the time-intensive process of generating high-volume images results in a trade-off that needs to be carefully balanced. This study aims to determine optimal instrument settings to generate representative images of pathological tissue using digital microscopy. Using various settings, an H&E stained sample was scanned using the ZEISS Axio Scan.Z1. Next, nucleus segmentation was performed on resulting images using StarDist. Subsequently, detections were compared between scans using a matching algorithm. Finally, nucleus-level information was compared between scans. Results indicated that while general matching percentages were high, similarity between information from replicates was relatively low. Additionally, settings resulting in longer scanning times and increased data volume did not increase similarity between replicates. In conclusion, the scan setting ultimately deemed optimal combined consistent and qualitative performance with low throughput time.

## Introduction

Traditional pathology, where biopsies of tissue samples are examined by the pathologist using light microscopy, is still implemented in many hospitals worldwide [1]. However, digital pathology is an emerging field with applications in research and clinical settings. In this field, digital slide scanners generate whole slide images (WSIs) of histopathological slides [2]. Moreover, these WSIs open up new possibilities for analysis using artificial intelligence (AI) and spatial statistics to acquire more information and aid in the diagnosis and prognosis of a patient [1–3].

In recent years, the medical field’s focus has shifted towards a tailored approach for determining treatment strategies, called precision medicine. This approach requires various medical sources such as a patient’s clinical history, genomics, and other pathological analyses. Digitization is paramount to integrate these medical sources and paves the way for digital pathology as a key player in this process [4].

Digital pathology creates a major window of opportunity for AI to improve a patient’s diagnosis [2, 4] significantly. The use of AI on WSIs can lead to a new field of discovery, where subclinical features beyond human perception can be used for advanced tasks such as further classification of the tumor microenvironment. One up-and-coming subclinical set of features is the morphological pattern of nuclei, which can be used to differentiate between malignant and benign breast tumors [5], estimate prostate cancer recurrence [6], or predict short-term and long-term survival [7].

When pathologists assess slides manually, a first inspection of the tissue is typically performed on low magnifications. After identifying specific regions of interest (ROIs), these areas are evaluated at higher magnifications to draw conclusive insights. This concept applies to both traditional and digital pathology. In higher magnifications, tissue, and cell properties become more visible, allowing for better quantification of the tissue in question. A clearer view of the tissue leads to a better patient diagnosis.

To enable the discovery of subclinical features using AI, the WSI quality must be equal or superior to what is seen under the microscope under different magnifications [8]. As digital pathology is generally still in its early stage, no standard with which all tissue slides are digitized is currently available. This leads to a considerable variation of WSI in terms of image resolution, magnification, scan consistency, focus, etc. Manual validation of every scanned tissue slide is very time-consuming and labor-intensive. Additionally, quality assessment can be subjective, depending on the lab and the scan operator. It is, therefore, necessary to study and discuss the effects of different scan settings on the tissue slides and their downstream analysis with AI and computer vision tools.

Generally, a scanner that creates WSIs has an optimal setting to ensure high quality. Still, there are drawbacks when implementing this setting in a workflow where many slides must be scanned. Two non-image-related drawbacks that are considered are time and data volume. Increasing quality will have repercussions concerning the time it takes to scan the image, as well as the size of the resulting data file. Both factors are essential when considering automation and further processing of the images.

Additionally, a higher data volume results in higher storage costs. To combat this, compression is widely used to reduce image size. However, this can result in image artifacts [9].

Several quality assessment tools for generated histopathological WSIs are available to optimize the scanning protocol, such as HistoQC and PathProfiler [10, 11]. These tools can identify common artifacts that may affect the quality of the WSIs. For example, images that contain scratches or folds, unfocused areas, or other expected quality losses must be removed from the dataset [12]. However, these tools do not assess the quality of an image in function of the performance of an AI model trained for the segmentation or classification of cell types.

Multiple elements may contribute to high-quality images for data analysis. Some steps that can be optimized include the focus point strategy, sharpness measurement set, single layer versus z-stack, and optionally compressing the data. A detailed description of these settings can be found in the Axio Scan.Z1 manual [13]. However, there is currently no golden standard protocol for digitization for all available tissue types and staining methods. Therefore, optimization of the scanning protocol is needed, especially when the goal is to apply AI analyses to these images.

This manuscript presents the optimization of a scanning protocol for tissue slide digitization. Hence, we aim to achieve a good balance between image quality on the one hand and the drawbacks of scanning time and file size on the other hand. The generation of high-quality images is prioritized, as the rejection of low-quality slides results in time delays due to rescanning or resampling.

Aside from measuring quality, we also focus on the inter-scan variability. Even when applying a set scan setting, results of the nucleus detection algorithm might differ slightly across different scans of the same sample, as the algorithm might make non-identical detections when pixels give slightly different information in two separate scans. These detections could lead to many high-quality but inconsistent images, which are unsuitable input data for machine learning and AI modeling techniques. Therefore, as the primary goal, detections should be as consistent as possible across fixed-setting scans while keeping scan quality high and scanning time acceptable for continuous generation of WSIs.

## Methods

### Human sample

To identify the optimal scan profile as an initial step, we used one representative biopsy/surgical resection (a cryo-fixated Hematoxylin and Eosin (H&E) stained tumor-free lymph node) from a non-small cell lung cancer patient who was diagnosed with invasive squamous cell carcinoma. For the validation of the chosen scan profile as a final step, five additional biopsies (Formalin-Fixed Paraffin-Embedded, H&E stained, tumor-containing tissue) were used from non-small cell lung cancer patients. The cases were selected from the prospective PROLUNG study and were obtained from the pathology department of Ziekenhuis Oost-Limburg, Belgium (Start of study: 28-05-2018, End of study: 31-12-2022). The recruitment period started at 29-05-2018 and ended at 20-01-2022. The initially selected biopsy was accessed for scanning and research purposes between 4-11-2021 and 05-02-2022 and the five biopsies used for validation were accessed between 01-12-2022 and 09-07-2024. Ethical approval was granted by *Comité Medische Ethiek Ziekenhuis Oost-Limburg* (18/0023U). Written informed consent was obtained from all patients. This tissue slide was selected based on its median tissue size. The tissue was stained with the automated tissue stainer "linear slide stainer ST4020" (Leica).

### Scan profile

A 40x brightfield scanning profile was created using the Axio Scan.Z1 (Zeiss) and its ZEN software (Zeiss). While generating the profile, several parameters were taken into account. The standard settings regarding the global overview, preview, and tissue detection standard settings were not altered during optimization. An automated tissue detection was performed using the standard settings and determining the best focus map settings.

First, the 10x objective lens was combined with a fixed number of focus points (4) strategy and basic sharpness measurement set in the coarse focus setting. Several strategies were explored to achieve the optimal focus across the entire sample. The 40x objective lens was used for the fine focus setting and the onionskin focus point strategy. For the latter, the density of the focus points and the maximum number of points were adapted. More specifically, the focus point density refers to the percentage of tiles covered with one specific focus point, which can be limited to a value set by the maximum number of points [13]. The following conditions were tested for the maximum number of focus points: 100, 150, and 200, combined with a density of either 5 or 10%. In addition, the "best" and "Hg^8 sharpness" measurement settings were used for comparison. An overview of the different focus map settings is provided in **Table 1**.

**Table 1:** Overview of the tested focus map settings. The reference standard was generated in combination with a Z-stack and Extended Depth of Focus (EDF).

*Setting name*	*Maximum number of focus points*	*Focus point density (in %)*	*Sharpness option*
**S1**	100	5	Best
**S2**	100	5	Hair gradient 2^8
**S3**	150	10	Hair gradient 2^8
**S4**	150	5	Best
**S5**	150	5	Hair gradient 2^8
**S6**	200	10	Hair gradient 2^8
**S7**	200	5	Best
**S8**	200	5	Hair gradient 2^8
**Reference standard**	24	5	Best

In concordance with the fine focus, a 40x objective lens was used in the final step. Moreover, several parameters were tested for compression to determine image quality. Lossless compression was chosen instead of lossy compression to retain as much information as possible for further downstream analysis. Single-layer scanning versus Z-stack scanning in combination with the Extended Depth of Focus (EDF) function was also compared. The latter was considered our optimal scan due to its extensive setting profile and scanning time, further referred to as the reference standard. Here, we used the standard maximum number of points with a 5% density in the fine focus map settings.

Hereafter, the complete scan profile will be referred to as the scan settings.

To validate the chosen setting, we used the default setting, which utilizes the 5x objective lens for the coarse focus, and 40x objective lens for the fine focus and final step. In the default profile, only one point of focus is selected for the image, and the sharpness option is set to “basic”.

### Nuclei detection

After scanning tissue slides, nuclei detection was performed to provide information for further downstream analyses. Scans were visualized in QuPath, an open-source bioimage analysis program [14]. As a first parameter to determine image quality, simple nucleus segmentation was performed using a state-of-the-art deep learning algorithm called StarDist [15]. The StarDist algorithm utilizes a pre-trained model using the MoNuSeg 2018 dataset [16]. To lower the algorithm’s execution time over multiple repeated images while maintaining sufficient information, ten ROIs were selected. Each region was 2,000 by 2,000 pixels, corresponding to 220 μm by 220 μm on the scans with a pixel size of 0.11 μm. In addition, the deep learning algorithm was applied to images with resolutions of 0.22 μm and 0.44 μm per pixel derived from the original image. The algorithm’s settings were kept the same for all ROIs to ensure maximum comparability. Slight location shifts of the tissue between different scans were observed, possibly due to a different selection procedure of the tissue of interest by the Axio Scan.Z1. To ensure consistency while comparing different scan settings, the tissue was matched via affine transformation of the image, thereby correctly aligning the nuclei within the chosen ROI. After detecting all nuclei in the ROI, several features of the nuclei were generated, including the location of the centroid of the nucleus, summary statistics of the H&E values in each pixel, and the area of the detected nucleus.

The number of detected nuclei was also used to measure the image quality, as higher-quality images should better preserve tissue features, leading to more accurate detections.

### Quantitative measurements

All nuclei present in the ten ROIs were annotated manually as a reference for the assessment of the different conditions quantitatively. Two biomedical scientists with histological expertise, hereafter referred to as HIS1 and HIS2, manually selected five additional 2000 by 2000 pixel ROIs in which the nuclei were annotated using the annotation tool in QuPath, followed by automatic segmentation using the algorithm described above. The automated detections and manual annotations were compared per region via overlay.

A similar manual assessment was performed to verify the nucleus detection algorithm’s efficiency. First, the detection was performed on the ten 2000 by 2000 pixel ROIs. Afterward, the detections made by StarDist were manually corrected and categorized into four classes: correct detections, nuclei not detected by the algorithm, detections that did not correspond to a nucleus, and detections considered incorrect due to under- or over segmentation by the algorithm. HIS1 and HIS2 manually corrected these segmentation errors by making a new annotation.

### Inter-scan variability

After scanning tissue slides, the optimal image quality was determined using inter-scan variability as a parameter. The inter-scan variability was assessed by verifying how often a unique nucleus was detected at a highly similar location in multiple scans using identical scan settings. Three technical replicates were made for each scan setting. Ten smaller ROIs within the sample were identified in every scan and compared.

An in-house image registration algorithm was developed to compare the same region across two different scans. First, the x- and y- coordinates of the centroid of each nucleus were kept as information. The data could then be seen as a point pattern where each event is a nucleus. Each nucleus corresponding to one point was then matched to a nucleus corresponding to the other point pattern, which was done by choosing its nearest neighbor of the other point pattern within a 10 μm x 10 μm square centered around the nucleus. Note that two nuclei could have the same nearest neighbor. In this case, those nuclei from the two-point patterns with the smallest distance between them are considered the correct match. The remaining nuclei with the same nearest neighbor remained *unmatched* (i.e., failed to have found a matching nucleus). These nuclei were considered for matching a second time in case a different potential match existed. After this second attempt, there were matched and unmatched nuclei from the initial batch for both point patterns. The percentages of matched and unmatched nuclei relative to the whole batch were used to indicate inter-scan variability. Small coordinate shifts could have occurred when overlaying two detections, which was solved by shifting one side towards the nearest neighbors of the other side.

As a result, a small percentage of nuclei from either side was no longer considered, as they lay beyond the corresponding area on the other side. These were noted as ’edged-out.’ A benchmark to compare the percentages as mentioned above was constructed. HIS1 and HIS2 both manually annotated the same five tissue regions. The matching algorithm was then used to compare both independent annotations. Furthermore, to inspect the algorithm’s general performance, HIS1 and HIS2 manually corrected the nucleus detections made by the algorithm. The same ten ROIs used for the analyses described in the methods section were reviewed (specifically on the second scan of the S7 setting, specified in Table 1).

A batch of matched nuclei was observed on either side per pairwise scan comparison. Each nucleus had descriptive information determined by the algorithm, as listed before. Therefore, we were able to analyze the agreement between different measurements. In this context, this translates to measuring how similar each nucleus’ aspect is compared to its match from another scan. Bland-Altman plots, also called difference plots, visualize the average measures of the matching nuclei versus the difference among them. Limits of agreement (LOAs) indicate how far apart the measures of the matching nuclei are and were used as indicators of inter-scan variability. For some variables (e.g., nucleus area), the measurements were heteroscedastic (i.e., differences become larger when measurements are larger).

Consequently, instead of computing the conventional difference between the two measurements, we chose to consider their ratios by log transforming first and subsequently computing the differences, the median, and LOAs of these differences, after which these values were back-transformed [17]. In addition, we chose the 2.5% and 97.5% quantile of the differences of log-transformed values to account for violating the normality assumption even after log-transformation of the data [17]. Finally, confidence intervals around the median and quantiles were constructed by bootstrapping (i.e., randomly sampling the data with replacement and recalculating the statistics in question) the data.

## Results

### Scan time and file size

The resulting scan time and file size differ considerably depending on the chosen scan setting. In addition to the quality of the scans, the scan time and file sizes were also considered. **Table 2** summarizes the average values of the three repeated scans concerning scan times and file sizes. A change in the maximum number of points slightly increased scan times but did not lead to larger file sizes. The density of the focus points and the chosen sharpness measurement set did not alter scan time or file size. Using the reference standard settings, the scan time heavily increased while the file size remained within the range of the other settings, which may be due to the EDF function used in this setting. Using a Z-stack without performing EDF resulted in a much larger file size. One scan with setting S7 has fewer focus points, slightly lowering the average scan time.

**Table 2:** Overview of the variables of interest regarding the scanned slides for the reference standard setting and the eight settings (n = 3) used. One of the three replicates in setting S7 contained 199 focus points instead of the expected 200, therefore the average number of points for that setting is 199.667.

Scan setting	Number of focus points used	Average scan time (min.)	File size (GB)
**S1**	100	20.7	29.35
**S2**	100	20.8	28.99
**S3**	150	24.2	28.55
**S4**	150	24.1	29.03
**S5**	150	24.0	28.64
**S6**	200	27.8	28.93
**S7**	199.667	27.7	29.25
**S8**	200	27.3	29.03
**Reference standard**	24	119.8	31.02

### Nuclei detection using StarDist

**Fig 1:**
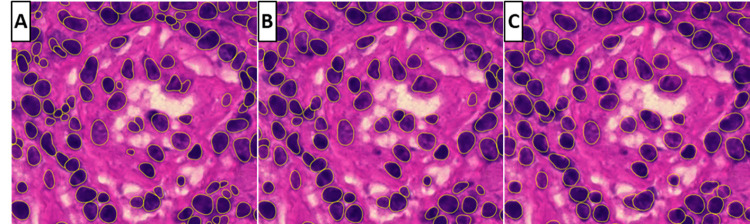
Three images with nuclei detections (yellow outline) using the StarDist segmentation tool. A: Detections on a resolution of 0.11 μm per pixel. B: Detections on a resolution of 0.22 μm per pixel. C: Detections on a resolution of 0.44 μm per pixel.

On each replicate of each scan setting, nucleus detection was performed on the ten ROIs mentioned above using the StarDist deep learning algorithm in QuPath. Additionally, three resolutions were considered. Fig 1 shows a side-by-side comparison of detections on a small section of one of the ten regions using three resolutions. To create this example, an image of the reference standard was used. Detections on Fig 1A are based on the highest resolution, while detections on Fig 1B and 1C are based on resolutions of 0.22 and 0.44 μm per pixel, respectively. In general, detections using the lowest resolution are less smooth and, therefore, do not have correct boundary alignment. Small nuclei are also left undetected in this resolution. While detections on the highest resolution have better boundaries on each nucleus, some larger nuclei are left out or segmented inadequately.

### Descriptive statistics for each scan setting

**Fig 2:**
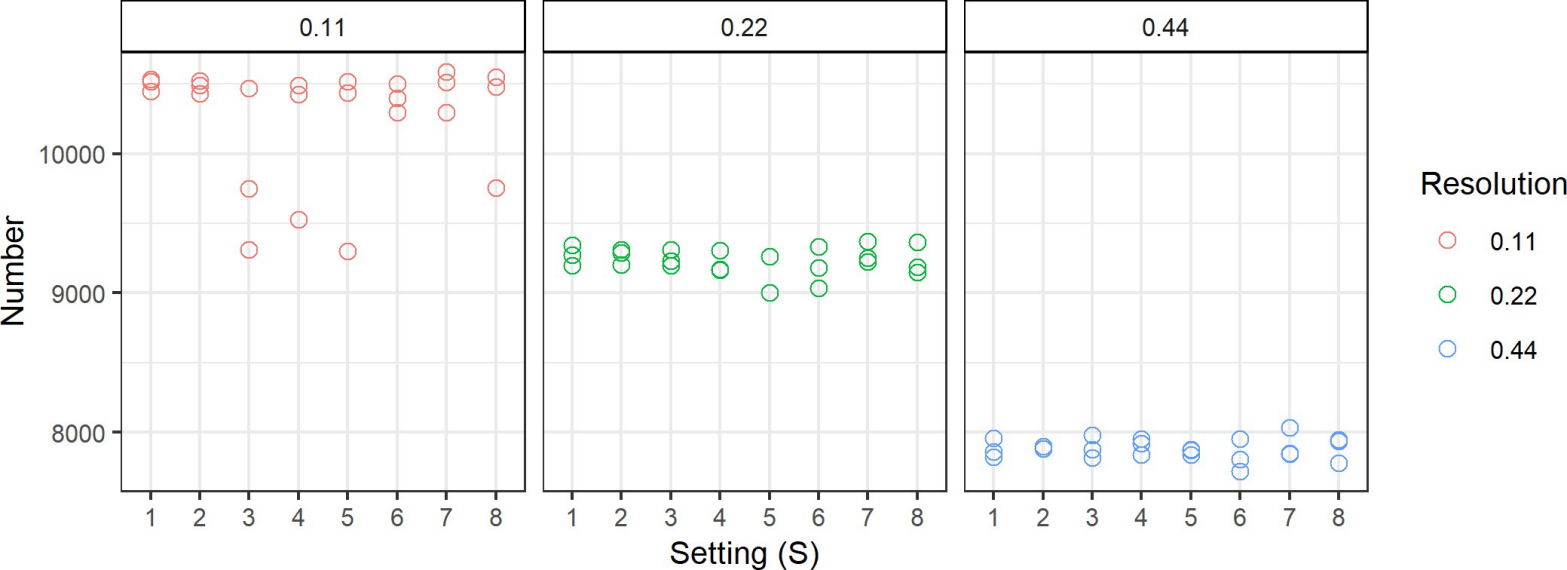
Number of detected nuclei per scan for each scan setting with AI detections on resolutions 0.11, 0.22, and 0.44 μm.

To assess inter-scan variability, we first calculated two summary statistics related to nucleus detection: mean number of nucleus detections and mean nucleus area. The number of nuclei detections per scan is presented in Fig 2. The mean and standard deviation of the nucleus area per nucleus, subdivided by resolution, are presented in S1 Table. This shows that the differences between scan settings were minimal, whereas differences between resolutions were more prominent. More specifically, the mean nucleus area increased, and the number of detected nuclei decreased as the resolution decreased.

**Fig 3:**
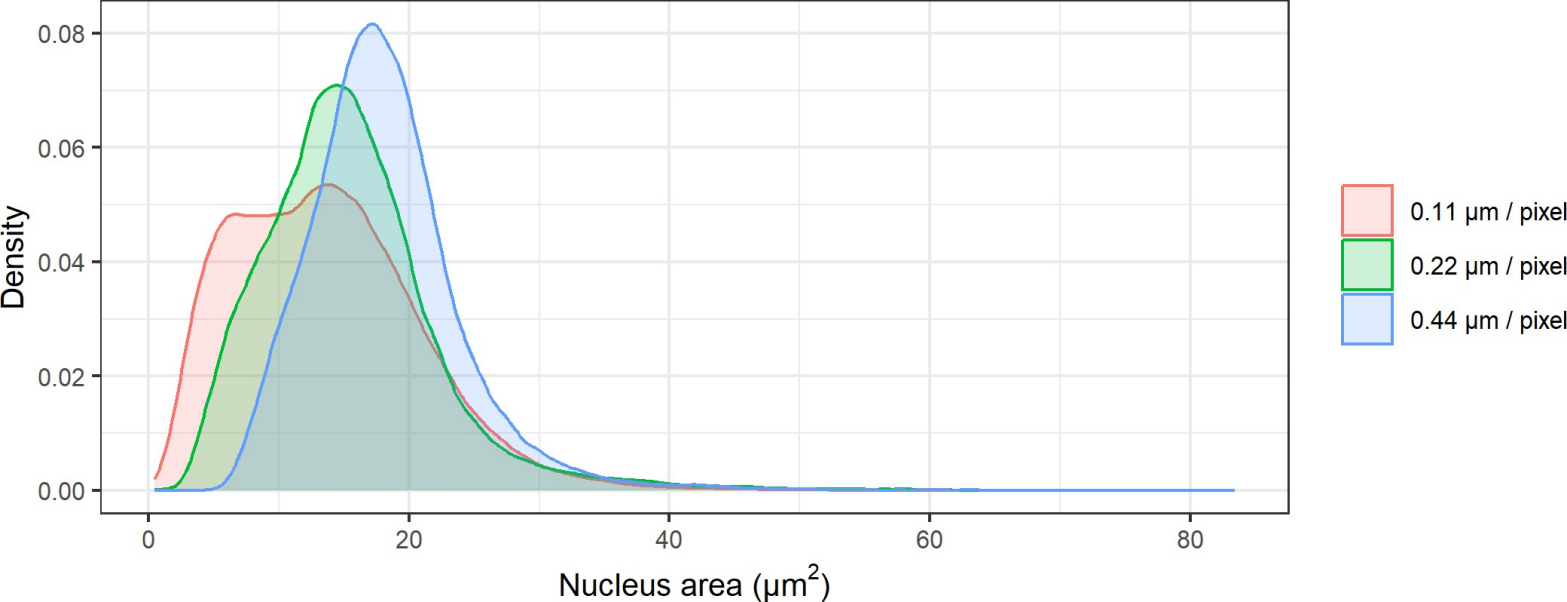
Density (probability per unit) of the variable nucleus area in μm^2^ across all included scans for the resolution settings 0.11, 0.22, and 0.44 μm per pixel.

The detailed distribution of the nucleus area in a given pixel setting is presented as a density plot (i.e., a smoothed visualization of the value distribution) in Fig 3. Resolution 0.11 shows two peaks in its distribution of nucleus areas with a high density of nucleus areas below 10 μm^2^, which was not seen for resolutions 0.22 and 0.44. Furthermore, a general rightward shift in distribution can be observed as the resolution decreases, resulting in an increasing distribution mode.

### Inter-scan variability

The matching percentages were determined for each scan setting. The benchmark for matching percentages by HIS1 and HIS2 yielded a matched, unmatched, and edged-out percentage of 90.25%, 9.71%, and 0.04%, respectively. In addition, the inspection by HIS1 and HIS2 showed that, over all regions, 20,349 nuclei (mean nucleus area of 13.46μm^2^) were detected by the algorithm, of which 194 (0.95% of total nuclei, mean nucleus area of 4.43μm^2^) were deemed not a nucleus by the reviewers. An additional 1,380 nuclei (6.78% of total nuclei, mean nucleus area of 16.00μm^2^) were manually detected but were missed by the algorithm. For 460 of the detected nuclei (2.26% of total nuclei, mean nucleus area of 14.21μm^2^), the detection was considered incorrect and was corrected by manual annotation. The correction resulted in the annotation of 522 nuclei. Therefore, the 460 detected nuclei (mean nucleus area of 19.62μm^2^) made up 88% of the true number of nuclei.

**Fig 4:**
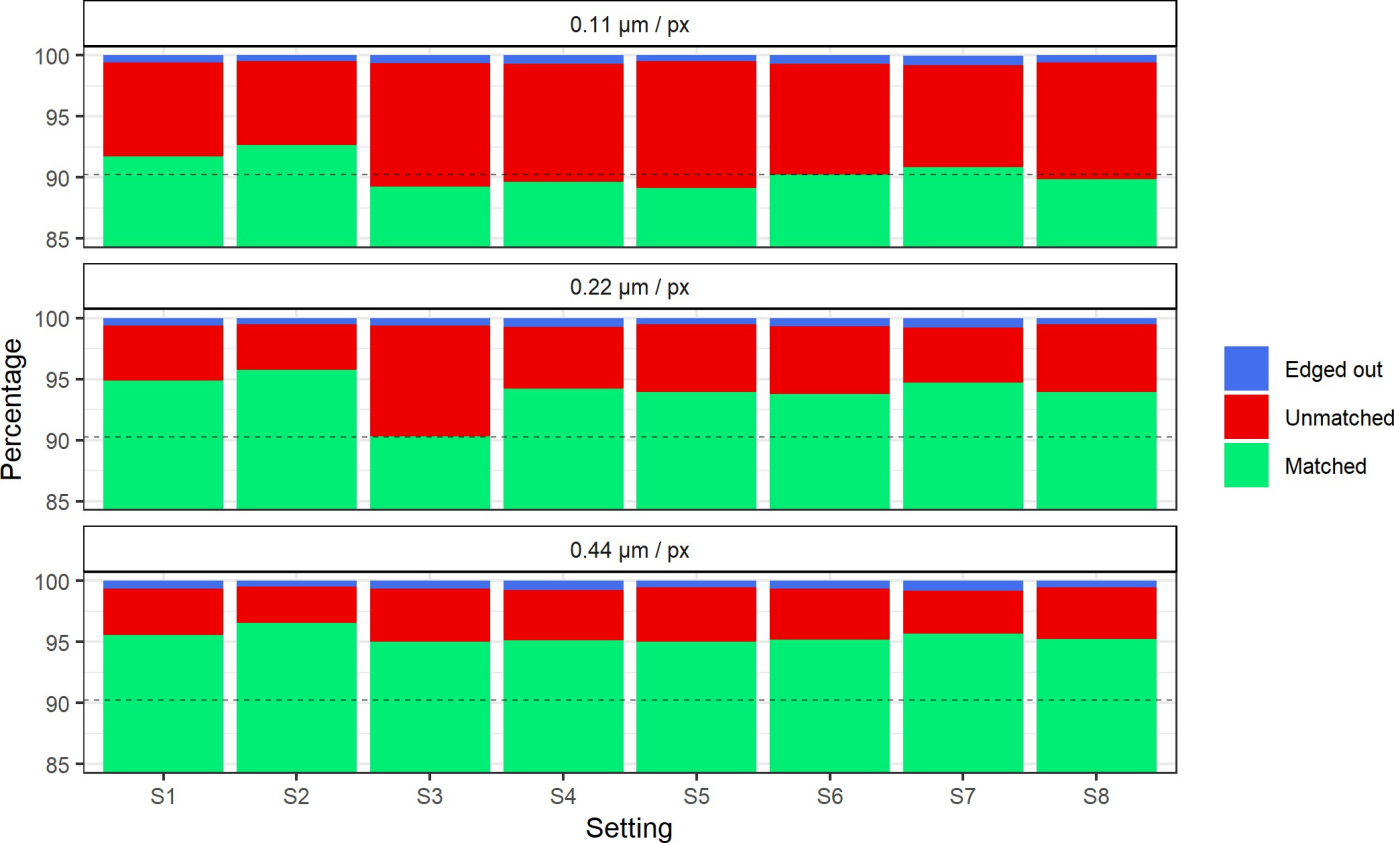
Matching percentages of pairwise comparisons within repeats of the same scan settings for resolutions of 0.11, 0.22, and 0.44 μm per pixel. The Y-axis is limited from 85 to 100% for visual purposes. The dashed black line indicates the benchmark-matched percentage calculated by comparing manual annotations. Setting names are detailed in Table 1.

The matching percentages for each setting per resolution are presented in Fig 4. For all resolutions, the S2 scan setting had the highest matching percentage. In all resolutions, the matching percentage for this setting was above the benchmark (90.25% of nuclei matched) established by matching manual annotations. The matching percentage again more prominently differed between resolutions than between scan settings. A decrease in the chosen resolution increased the matching percentage, independent of the scan setting.

**Fig 5:**
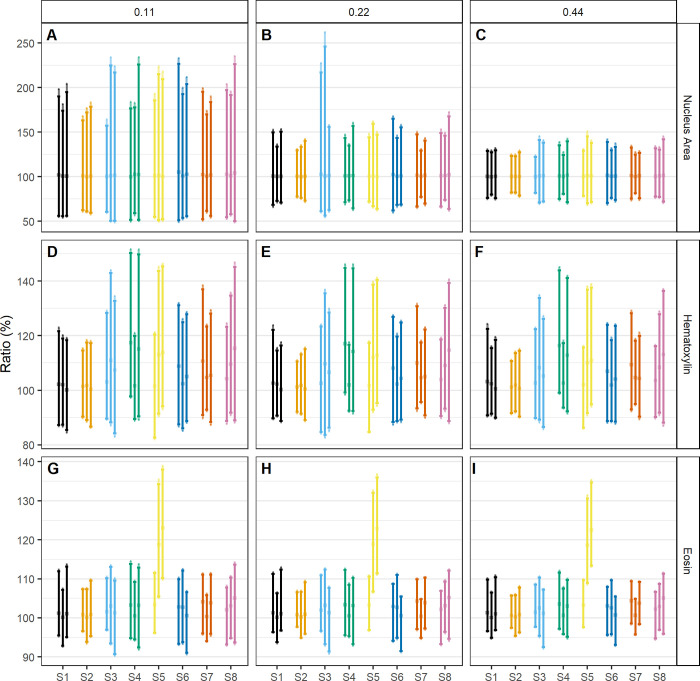
Limits of agreement between matched nuclei of pairwise comparisons within repeats of the same scan settings (S1-8). LOAs are shown on a ratio scale (in %). (A) shows the nucleus area for 0.11μm/pixel resolution, (B) for 0.22μm/pixel, and (C) for 0.44μm/pixel. The median hematoxylin value of the nucleus is shown in (D) for 0.11μm/pixel, (E) for 0.22, and (F) for 0.44. The median eosin value of the nucleus is shown in (G) for 0.11μm/pixel, (H) for 0.22, and (I) for 0.44. Comparisons are shown in order of scan 1 vs. 2, scan 1 vs. 3, and scan 2 vs. 3 within each setting. Confidence intervals around the median, 2.5%, and 97.5% quantiles were built as a bootstrapped percentile interval. Setting names are detailed in Table 1.

The LOAs, which indicate how much difference is found between matched nucleus measures, and their bootstrap percentile intervals are shown in Fig 5. None of the settings displayed a consistently smaller LOA compared to other settings. However, the setting previously noted to have the best matching percentages (S2, as coined in Table 1) is consistent among the groups with smaller agreement intervals. The differences in widths of the LOAs are larger between resolutions than between scan settings, which is in line with our previous results.

The LOAs concerning the median H&E values of the nucleus are also shown in Fig 5. Again, there is no setting with a consistently smaller LOA, but those belonging to the aforementioned best-matching setting are among the smaller LOAs.

### Optimal scan setting validation

Matching percentages for the chosen optimal setting in comparison to the default setting are shown in S2 Fig. In four out of five samples, the chosen setting had a higher matching percentage than the default setting. All matching percentages for the chosen setting were superior to the aforementioned manual benchmark of 90.25%, whereas for one sample, the default setting attained a matching percentage under the benchmark (88.28%).

LOAs for the chosen setting versus the default setting are shown in S3 Fig. Consistency in nucleus area measurements was relatively similar in both settings with the exception of one sample, in which the default setting had less consistent measurements. For hematoxylin values, three samples showed similar LOAs for both settings, one was more consistent in the chosen setting and one was more consistent in the default setting. Finally, for eosin values, in three samples, the default setting was relatively more consistent than the chosen setting. In the remaining two samples, both settings yielded similar consistency in one sample and the chosen setting showed more consistency in the other.

## Discussion

In this study, we explored design methods to select the optimal setting for the generation of high-quality WSI using digital microscopy via an optimization procedure. The main factors that aided our decision were inter-scan variability and quality of nucleus detection via a segmentation model based on AI. The goal was to obtain a scan setting that provided images with consistent segmentation results and minimal artifacts present while optimizing two non-image-related limiting factors: time and data volume. An additional result of a consistent scan setting was to ensure that few or no slides needed to be rescanned, minimizing the time lost in routine analyses. This added quality also aids computer vision models, which prefer higher-quality data than the human eye, to notice tissue features that an expert can ignore [18, 19].

### Throughput time as a trade-off aspect

One of the key parameters while implementing a digital pathology workflow is throughput times. Efficient scanner usage time yields more data to train algorithms to support diagnostics. By reducing scan times, faster image analysis becomes feasible, leading to an increased likelihood of reporting biomarker results promptly. Previous research highlights the significance of timely reporting in delivering high-quality clinical care to non-small-cell lung cancer patients [20]. In this context, extremely long scan times, such as those belonging to the previously labeled ’reference standard’ scanning type, are unfavorable. Even a slight increase in scan time by increasing the maximum focus points can add up quickly in an automated workflow. Hence, it is essential to assess whether increased scan times yield improved outcomes concerning the quality and consistency of detection.

### Algorithm performance and consistency

Three resolutions were considered for nucleus detection via the StarDist algorithm. In the lowest resolution of 0.44 μm per pixel, nuclei boundaries were not segmented adequately, leading to a loss in accuracy. Higher resolutions have good segmentation boundaries, but some large nuclei are only partially segmented on the highest resolution of 0.11 μm per pixel. This finding is related to the resolution of the images used to train the StarDist algorithm. The model is trained on the MoNuSeg 2018 dataset, which uses annotated data extracted from The Cancer Genome Atlas (TCGA) tissue slides with a corresponding approximated pixel size of 0.25 μm [16]. The dataset was also acquired using a 40x objective, but additional information is lacking. As a result, an increase or decrease in resolution of a factor of two will yield slightly different results after segmentation.

In addition, a lower resolution yielded a more significant matching percentage and agreement regarding inter-scan variability. However, the image quality and subsequent detections using a resolution of 0.44 μm were considered suboptimal. Lower resolutions lead to a decrease in the number of detected nuclei. However, the detected nuclei had a larger area.

The decreasing resolution indicated a general upward shift of the nucleus area (Fig 3). A nucleus detected by the algorithm will have an expected area based on its number of pixels. A higher-resolution image will have more pixels per nucleus, while an image of lower resolution will have fewer pixels per nucleus. This can yield detected nucleus areas that are shifted upwards for low-resolution images and shifted downwards for high-resolution images.

As a result, detections became more homogeneous, which increased the likelihood of finding a match and having similar traits to that match. This points to a limitation in using these measures to choose the optimal scan setting, as in this case, consistency between images did not point to quality but to loss of information. Additionally, the highest resolution yields a second peak in the left side of the distribution of the nucleus area, suggesting an increase in artifacts, i.e., small, color-intensive spots that will be falsely recognized as nuclei.

Finally, based on the matching percentages over all resolutions, scan setting S2 performed the best. However, no specific scan setting should be chosen when comparing the LOAs between measurements of the nucleus area because of lower inter-scan variability. Even the lowest matching percentages were not far below the provided benchmark. The assertion that time-intensive scanning methods are unsuitable in a clinical context is particularly strengthened by the above analyses, which do not show a clear relationship between an increased throughput time and image quality or inter-scan variability.

Considering all the abovementioned factors, scan setting S2 provides a good balance, was chosen as optimal scan setting and will be used for further analyses. The analysis for the validation of this setting by comparing it to a default setting showed that matching percentages were in most cases superior for the chosen setting. When comparing the consistency using LOAs, the chosen setting was superior for nucleus area values, was relatively similar for hematoxylin values, and was inferior for eosin values. Considering that all measurements were taken from the nucleus of each cell while eosin is inherently more relevant in cytoplasm, we consider eosin to be the least important of the three variables. As a result, we could interpret the consistency of the chosen setting to be superior.

The scanning resolution of 0.11 μm per pixel is chosen for two reasons. First, images scanned on this resolution will still have the ability to be downsampled into lower resolutions when necessary for algorithmic purposes. Second, scanned images will eventually be used as training samples to develop new deep-learning algorithms for digital pathology. This field will continue to improve, so higher-resolution images will eventually be generated. Resolution-based artifacts such as the wrongly segmented small spots mentioned before will be reduced with an increasing amount of annotated training data of higher resolution. It is therefore necessary to adapt to the ever-growing field of machine learning as quickly as possible.

By showing our optimization process, we hope to provide a guideline for future WSI generation, especially when downstream analysis using computer vision models will be performed.

### Limitations and future research

Scientific reports on the impact of image quality on the performance of deep learning and segmentation techniques are relatively scarce, with only a few papers available on this subject [18, 21, 22]. Therefore, our study aims to contribute to establishing a robust methodology for optimizing tissue scans in digital pathology. However, it is essential to acknowledge certain limitations inherent in this paper.

In our analyses, only three technical replicates were used per scan setting. This resulted in only three possible comparisons to the reference standard per set. Similarly, only three possible pairwise comparisons within a setting could be used. Each scan was therefore used in two out of three comparisons. Thus, even one suboptimal scan would have influenced the calculations and subsequent analyses. This is the case for one scan made with the S5 setting. As shown in Figs 4 and 5, LOAs concerning compared values of eosin of S5 scans were greater than 100%, indicating a general shift in color during the scanning process, which was confirmed through variable distribution inspection. In addition, a key assumption in the method presented above is the assumption of lack of interaction between disease-image features and the nucleus detection algorithm. In practice, though, the specific properties of the used nucleus detection algorithm potentially influenced our findings.

Our methods did not include a full factorial design, which would have included other factors such as artifact detection, image compression, and more exploration of the settings from the Zeiss Axioscan. Future research could include a complete list of settings or, if accessible, images produced by other scanners. Adding more images in the analysis workflow would also increase the generalizability of the results. Our analyses are limited to H&E-stained lung tissue, whereas similar analyses can be performed on fluorescent- or other immunohistochemical stainings.

## Conclusion

In this study, we presented a method for comparing instrument settings to scan and digitize pathological tissue consistently and qualitatively. With a list of possible scan settings, replicate images of the same tissue were made, and nuclei on these images were detected and subsequently compared between replicates in a measurement agreement framework. Finally, one scan setting was deemed optimal because of its consistency shown within this framework, as well as its favorable throughput time. Within the developing field of digital pathology, we are convinced this paper contributes to the rigorous investigation of the importance of image quality of pathological tissue.

## Supporting information

S1 Table Mean and standard deviation of nucleus area of detected nuclei (in μm) per scan for each scan setting with AI detections on resolutions 0.11, 0.22, and 0.44 μm. S1-8 refer to the settings mentioned in the manuscript.
(DOCX)

S1 Fig Limits of agreement between matched nuclei of pairwise comparisons between any scan (settings S1-8) and the ’reference standard’ scan. LOAs are shown on a ratio scale (in %). (A) shows nucleus area for 0.11μm/pixel resolution, (B) for 0.22μm/pixel, and (C) for 0.44μm/pixel. The median hematoxylin value of the nucleus is shown in (D) for 0.11μm/pixel, (E) for 0.22, and (F) for 0.44. The median eosin value of the nucleus is shown in (G) for 0.11μm/pixel, (H) for 0.22, and (I) for 0.44. Comparisons to the reference standard are shown in order of scans 1, 2, and 3 of a given setting, measuring their likeness to this reference standard. Confidence intervals around the median, 2.5%, and 97.5% quantiles were built as a bootstrapped percentile interval. No setting with a consistently smaller agreement interval could be found, though slight differences in settings could still be noted. Wider LOA’s were found for scans using the 0.44 μm/pixel resolution, as the reference standard is created at a 0.11μm/pixel resolution. Matched nuclei will therefore have a larger area in the low resolution scans, resulting in a larger discrepancy. Setting names are detailed in Table 1.
(TIF)

S2 Fig Matching percentages of pairwise comparisons within repeats of the same scan settings for D (default setting) and C (chosen optimal setting). All cells were divided into those who found a match in the comparison (matched), those who did not (unmatched) and those not considered due to lying in non-overlapping areas (edged out). Samples 1–5 are shown separately. The Y-axis is limited from 87.5% to 100% for visual purposes. The dashed black line indicates the benchmark-matched percentage calculated by comparing manual annotations.
(TIF)

S3 Fig Limits of agreement between matched nuclei of pairwise comparisons within repeats of D (default setting) and C (chosen optimal setting). LOAs are shown on a ratio scale (in %). (A-E) show nucleus area LOAs for samples 1 through 5 respectively. The median hematoxylin value of the nucleus is shown in (F-J) for samples 1 through 5 respectively. The median eosin value of the nucleus is shown in (K-O) for samples 1 through 5 respectively. Comparisons from left to right: scan 1 vs. 2, scan 1 vs. 3, and scan 2 vs. 3 within each setting. Confidence intervals around the median, 2.5%, and 97.5% quantiles were built as a bootstrapped percentile interval.
(TIF)

## List of abbreviations

AI: Artificial intelligence
EDF: Extended depth of focus
H&E: Hematoxylin and eosin
LOA: Limit of agreement
ROI: Region of interest
WSI: Whole slide image
