# Supplementary material for: Optimization of whole slide imaging scan settings for computer vision using human lung cancer tissue
Source: PLoS One. 2024 Sep 9;19(9):e0309740. doi: 10.1371/journal.pone.0309740 (PMC11383235; doi:10.1371/journal.pone.0309740)
Supplement: S1 Table — S1-8 refer to the settings mentioned in the manuscript. (DOCX) [file pone.0309740.s001.docx]

**S1 Table: Mean and standard deviation of nucleus area of detected nuclei (in µm) per scan for each scan setting with AI detections on resolutions 0.11, 0.22, and 0.44 µm.** S1-8 refer to the settings mentioned in the manuscript.

| **Nucleus area (in** µm^2^ **)** | | | | | | |
| --- | --- | --- | --- | --- | --- | --- |
| **Resolution** | **0.11** | | **0.22** | | **0.44** | |
| **Scan settings** | **Mean** | **St.dev.** | **Mean** | **St.dev.** | **Mean** | **St.dev.** |
| **S1** | 13,49 | 7.20 | 15,41 | 6.89 | 18,10 | 5.94 |
| **S2** | 13,58 | 7.28 | 15,46 | 6.92 | 18,14 | 5.93 |
| **S3** | 14,02 | 7.06 | 15,18 | 6.90 | 18,19 | 5.93 |
| **S4** | 13,78 | 7.12 | 15,51 | 6.84 | 18,12 | 5.92 |
| **S5** | 13,80 | 7.17 | 15,52 | 6.84 | 18,17 | 5.94 |
| **S6** | 13,47 | 7.16 | 15,45 | 6.93 | 18,15 | 5.97 |
| **S7** | 13,54 | 7.18 | 15,43 | 6.90 | 18,08 | 5.91 |
| **S8** | 13,60 | 7.17 | 15,42 | 6.88 | 18,13 | 5.96 |
