# Supplementary figures and images for: Optimization of whole slide imaging scan settings for computer vision using human lung cancer tissue
Source: PLoS One. 2024 Sep 9;19(9):e0309740. doi: 10.1371/journal.pone.0309740 (PMC11383235; doi:10.1371/journal.pone.0309740)

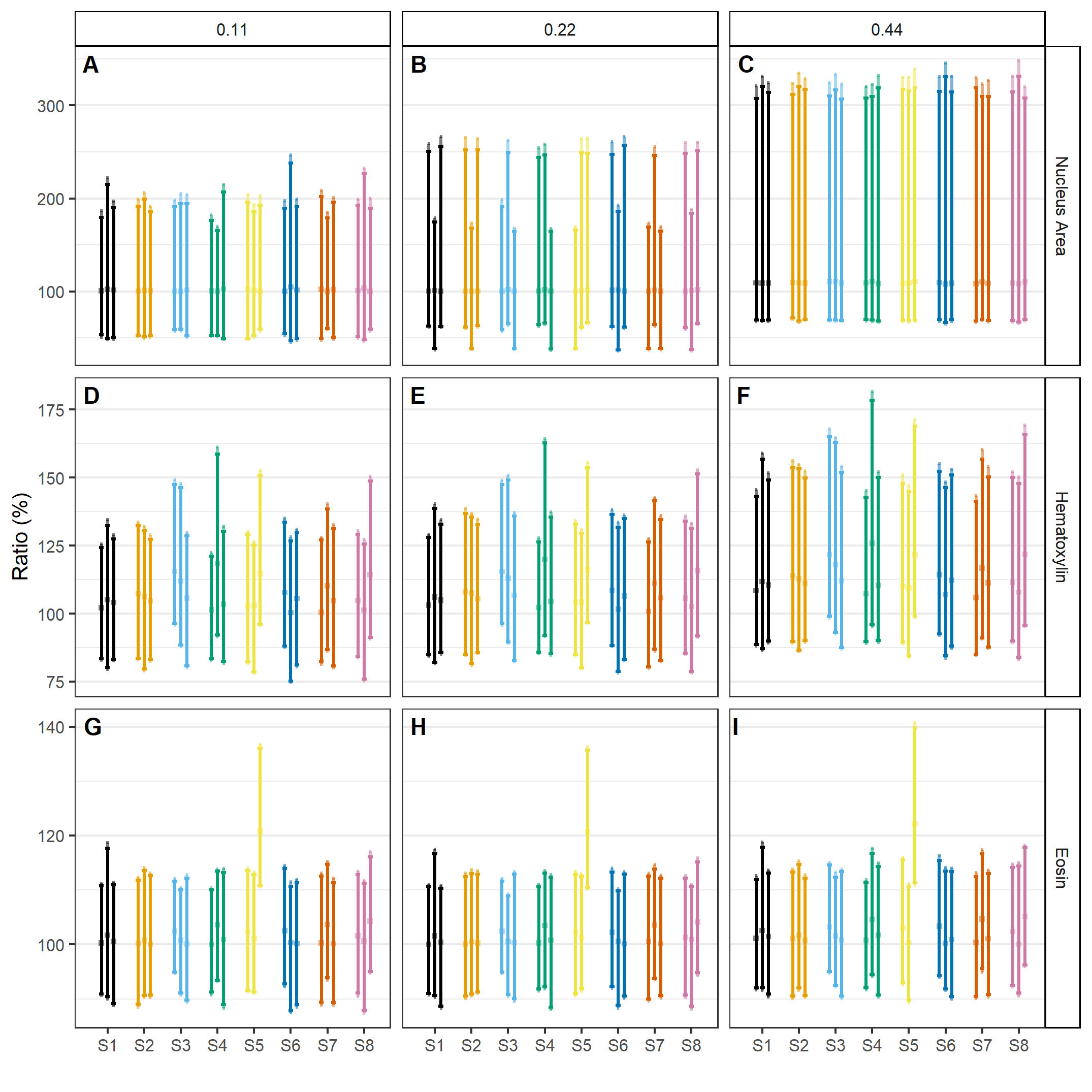

Supplement: S1 Fig — LOAs are shown on a ratio scale (in %). (A) shows nucleus area for 0.11μm/pixel resolution, (B) for 0.22μm/pixel, and (C) for 0.44μm/pixel. The median hematoxylin value of the nucleus is shown in (D) for 0.11μm/pixel, (E) for 0.22, and (F) for 0.44. The median eosin value of the nucleus is shown in (G) for 0.11μm/pixel, (H) for 0.22, and (I) for 0.44. Comparisons to the reference standard are shown in order of scans 1, 2, and 3 of a given setting, measuring their likeness to this reference standard. Confidence intervals around the median, 2.5%, and 97.5% quantiles were built as a bootstrapped percentile interval. No setting with a consistently smaller agreement interval could be found, though slight differences in settings could still be noted. Wider LOA’s were found for scans using the 0.44 μm/pixel resolution, as the reference standard is created at a 0.11μm/pixel resolution. Matched nuclei will therefore have a larger area in the low resolution scans, resulting in a larger discrepancy. Setting names are detailed in Table 1. (TIF) [file pone.0309740.s002.tif]

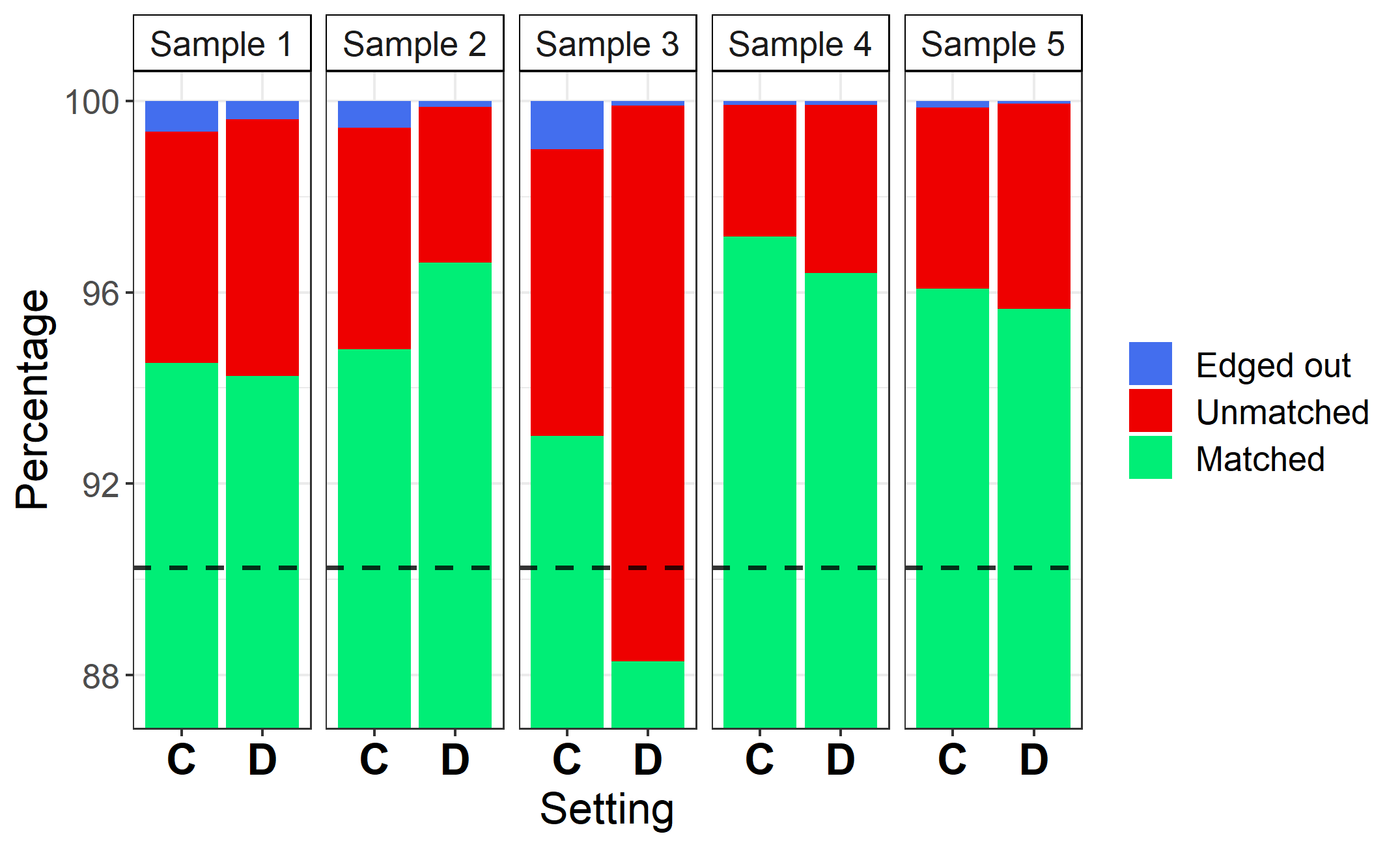

Supplement: S2 Fig — All cells were divided into those who found a match in the comparison (matched), those who did not (unmatched) and those not considered due to lying in non-overlapping areas (edged out). Samples 1–5 are shown separately. The Y-axis is limited from 87.5% to 100% for visual purposes. The dashed black line indicates the benchmark-matched percentage calculated by comparing manual annotations. (TIF) [file pone.0309740.s003.tif]

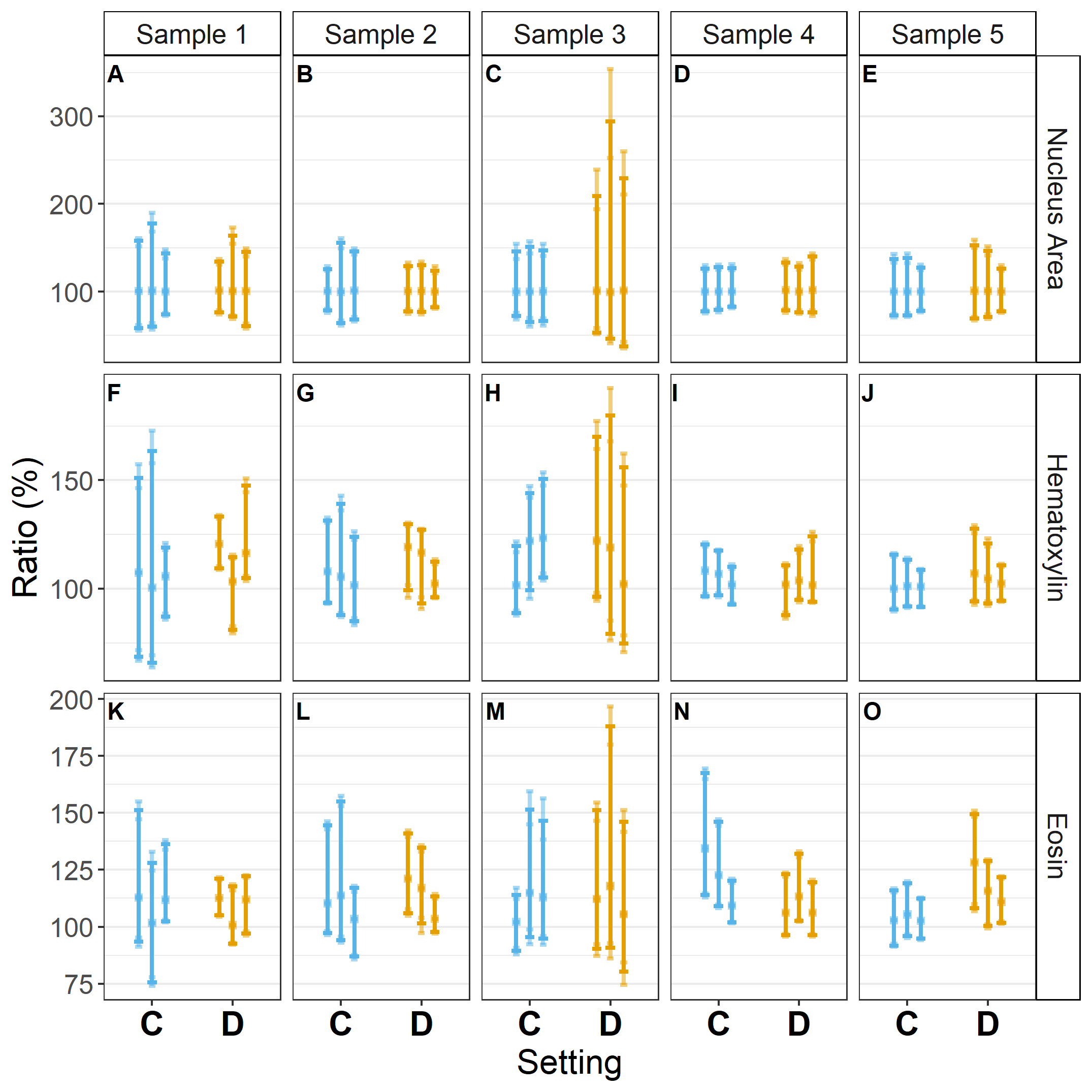

Supplement: S3 Fig — LOAs are shown on a ratio scale (in %). (A-E) show nucleus area LOAs for samples 1 through 5 respectively. The median hematoxylin value of the nucleus is shown in (F-J) for samples 1 through 5 respectively. The median eosin value of the nucleus is shown in (K-O) for samples 1 through 5 respectively. Comparisons from left to right: scan 1 vs. 2, scan 1 vs. 3, and scan 2 vs. 3 within each setting. Confidence intervals around the median, 2.5%, and 97.5% quantiles were built as a bootstrapped percentile interval. (TIF) [file pone.0309740.s004.tif]
